# How do patients with laryngeal cancer perceive their quality of life following total laryngectomy? Insights from a Tunisian cross-sectional study

**DOI:** 10.1192/j.eurpsy.2025.1776

**Published:** 2025-08-26

**Authors:** L. S. Chaibi, A. Amri, S. Hallit, M. Cheour, O. Feki, F. Fekih-Romdhane

**Affiliations:** 1 Department of Psychiatry, Razi Hospital, Mannouba; 2 Head and Neck Carcinologic Surgery, Salah Azaiez Institute, Tunis, Tunisia; 3 School of Medicine and Medical Sciences, Holy Spirit University of Kaslik, Jounieh, Lebanon; 4 Department of Psychiatry, University Tunis El Manar, Mannouba, Tunisia

## Abstract

**Introduction:**

Quality of life is a subjective evaluation that individuals make of the various aspects of their lives in relation to their health. Head and neck cancers and their surgical treatments, such as total laryngectomy (TL), change some of the most basic and important vital functions and can affect patients’ lives in many ways. The patient’s altered appearance, loss of their normally used voice, difficulty swallowing, and certain complications from this kind of surgery, all contribute to impaired quality of life by imposing daily limitations. Despite these considerable impacts, there is no or only very limited research addressing the quality of life of patients post- TL, highlighting the need for further exploration into this critical aspect of patient care.

**Objectives:**

The purpose of this study was to assess self-perceived quality of life in Tunisian male patients who underwent TL for laryngeal cancer.

**Methods:**

A descriptive cross-sectional study was conducted in the Head and Neck Carcinologic Surgery Department at Salah Azaiez Institute. Socio-demographic and clinical data were gathered. All patients completed the European Organization for Research and Treatment of Cancer Quality of Life Questionnaire Head and Neck Module (EORTC QLQ-H&N35), Depression, Anxiety and Stress scale, and Voice Handicap Index.

**Results:**

The study involved 30 male participants with a mean age of 62 years (±10 years). The mean EORTC QLQ-H&N35 total score was 60 ± 9.8. The areas of EORTC QLQ- H&N35 score most affected are shown in Figure 1.

Our results indicated that younger patients (P=0.002) and those from rural areas (P=0.04) tended to report better quality of life scores. Additionally, higher socioeconomic status was linked to a reduced quality of life (P=0.006). Patients who were classified as (N+) according to the Tumor, node and metastasis (TNM) staging, showed significantly better quality of life (P=0.004).A higher quality of life was significantly correlated with primary TL (P=0.004), while a lower quality of life was significantly associated with TL followed by radio-chemotherapy (P=0.005).Depression, anxiety, and stress were significantly correlated with poorer quality of life (P=0.002, P=0.001, P=0.001, respectively). Finally, lower quality of life scores were strongly associated with the Voice Handicap Index score (P=0.0001).

**
Figure 1.** Distribution of patients according to their EORTC QLQ- H&N35 scores.

**Image 1:**

**Figure:**
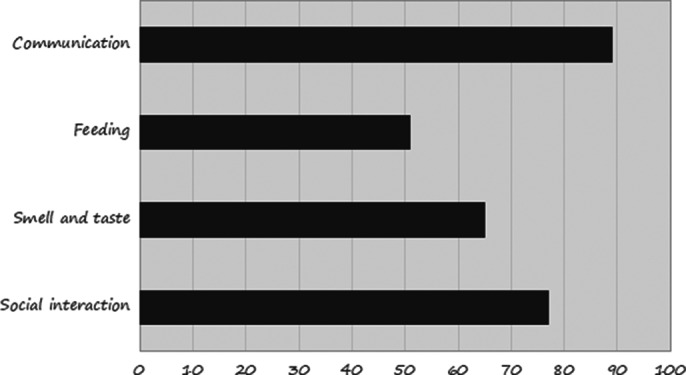

**Conclusions:**

In conclusion, patients who underwent TL for laryngeal cancer seem to exhibit an impaired quality of life, with factors like age, socioeconomic status, treatment type, voice handicap and psychological distress likely playing an important role. These findings underscore the need for comprehensive post-surgical care aiming at improving quality of life for people affected by this condition.

**Disclosure of Interest:**

None Declared

